# Balloon pulmonary angioplasty with inotropic agent and pulmonary vasodilators for chronic thromboembolic pulmonary hypertension

**DOI:** 10.1093/ehjcr/ytaf153

**Published:** 2025-03-26

**Authors:** Yuki Koga, Nobuhiro Tahara, Yoshihiro Fukumoto

**Affiliations:** Division of Cardiovascular Medicine, Department of Medicine, Kurume University School of Medicine, 67 Asahi-machi, Kurume 830-0011, Japan; Division of Cardiovascular Medicine, Department of Medicine, Kurume University School of Medicine, 67 Asahi-machi, Kurume 830-0011, Japan; Division of Cardiovascular Medicine, Department of Medicine, Kurume University School of Medicine, 67 Asahi-machi, Kurume 830-0011, Japan

**Keywords:** Balloon pulmonary angioplasty, Chronic thromboembolic pulmonary hypertension, PDE-III inhibitor, Pulmonary vasodilators

A 65-year-old female with chronic thromboembolic pulmonary hypertension (CTEPH) was referred to our hospital for balloon pulmonary angioplasty (BPA). The patient had presented with exertional dyspnoea and chest pain 2 years earlier and diagnosed with CTEPH in a referral hospital. On admission, the patient was in World Health Organization Functional Class (WHO-FC) III. Electrocardiographic and echocardiographic right ventricular (RV) overload and hypertrophy were not improved after a 3-month anticoagulation therapy with edoxaban of 60 mg once a day (*Figure 1* Panels A1, B1, and C1). Lung perfusion scintigraphy indicated multiple wedge-shaped defects (*Figure 1*; Panel D1). Right heart catheterization revealed pre-capillary pulmonary hypertension with a pulmonary artery pressure (PAP) of 97/35 mmHg with a mean pressure of 54 mmHg, pulmonary arterial wedge pressure (PAWP) of 11 mmHg, cardiac index (CI) of 1.95 L/min/m^2^, and pulmonary vascular resistance (PVR) of 13.4 Wood units. Pulmonary angiography (PAG) showed evidence of multiple embolisms in bilateral segments (*Figure 1*; Panel E1).

**Figure 1:**
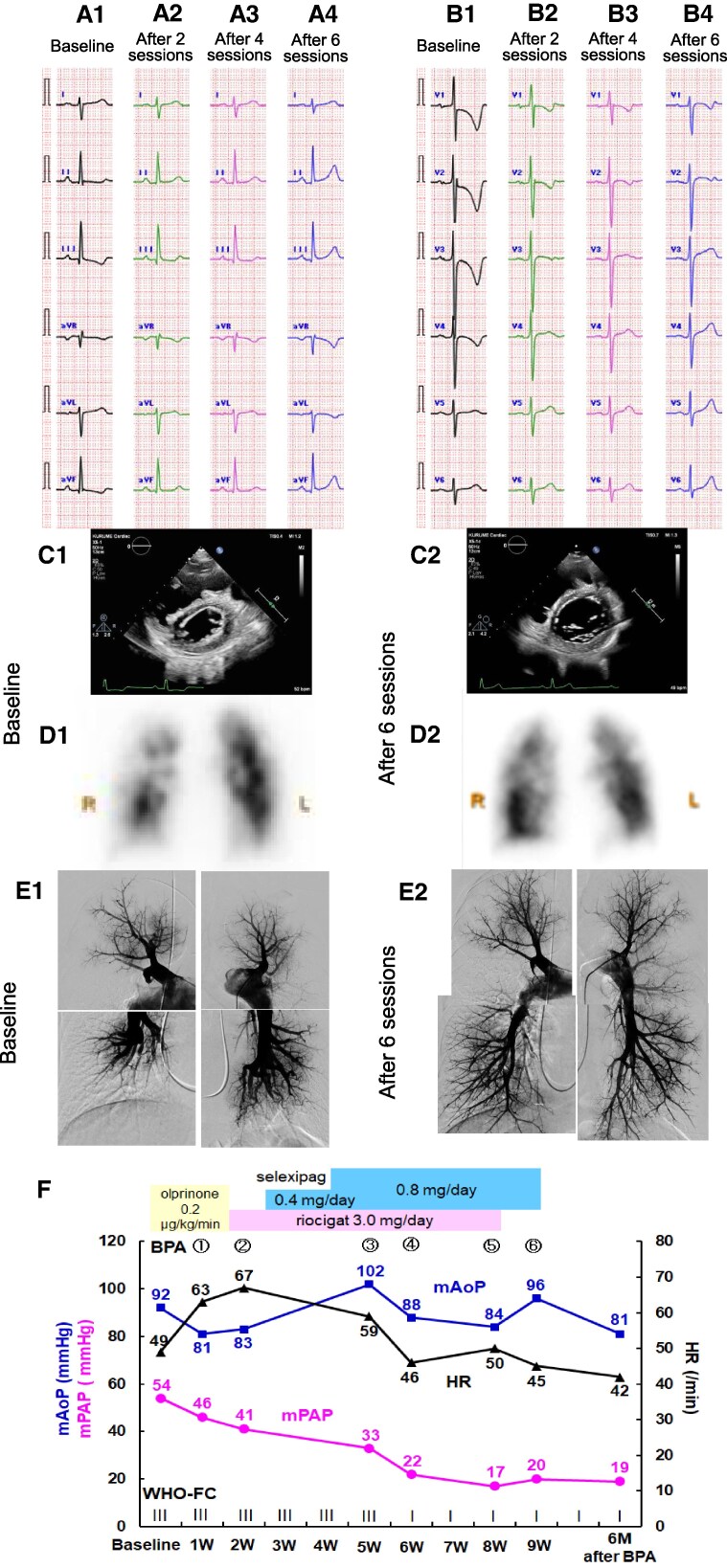
Clinical course of the patient. *(A, B)* Electrocardiographic right ventricular hypertrophy gradually resolved following balloon pulmonary angioplasty procedures. *(C)* An echocardiographic D-shape in the short-axis view at baseline is improved after six balloon pulmonary angioplasty sessions. *(D)* Lung perfusion scintigraphy indicates multiple perfusion defects in the bilateral lung at baseline and residual perfusion defects after six balloon pulmonary angioplasty sessions. *(E)* Pulmonary angiography shows improved bilateral pulmonary arterial flow following six balloon pulmonary angioplasty sessions. *(F)* Changes in haemodynamic parameters.

Our heart team decided to perform BPA for organized thrombotic lesions of the peripheral pulmonary arteries under the drip intravenous infusion of olprinone, a specific phosphodiesterase (PDE)-III inhibitor due to a low CI and high PVR. One day after the drip intravenous infusion of olprinone (0.2 μg/kg/min), PAP decreased to 84/29 mmHg with a mean PAP of 46 mmHg. First BPA was performed without complications under intravenous olprinone. After the first BPA procedure, olprinone was discontinued and riociguat was initiated at 3.0 mg/day (1.0 mg three times a day). Five days later, PAP decreased to 66/25 mmHg with a mean PAP of 41 mmHg and no riociguat-induced hypotension (*Figure 1*; Pane F). Selexipag was initiated at 0.4 mg/day (0.2 mg twice a day) after the second BPA and titrated up to 0.8 mg/day (0.4 mg twice a day) 1 week later. Under oral administration of 3.0 mg/day riociguat and 0.8 mg/day selexipag, third BPA was performed 4 weeks after the initial BPA, and fourth BPA 5 days later. Riociguat was discontinued after the fifth BPA and selexipag after the sixth BPA. Finally, six BPA sessions were performed for 19 segments on bilateral pulmonary arteries without complications, resulting in haemodynamic normalization (*Figure 1*; Pane F). Her 6-min walk distance was increased from 256 to 360 m, and N-terminal fragment brain natriuretic peptides level was decreased from 1141.7 to 60.5 pg/mL. Serial electrocardiography and echocardiography demonstrated a gradual resolution of RV overload and RV hypertrophy (*Figure 1*; Panels A2-4, B2-4, and C2). ST-segment and T-wave changes in the inferior leads were improved faster than those in the precordial leads. Although serial lung perfusion scintigraphy revealed multiple residual defects (*Figure 1*; Panel D2), a drastically haemodynamic change was seen with a mean PAP of 19 mmHg, and PVR of 2.4 Wood units. Bilateral pulmonary arterial flow was improved up to the peripheral pulmonary arteries (*Figure 1*; Panel E2). At follow-up 6 months after the last BPA, symptoms had completely resolved without pulmonary vasodilators (WHO-FC I). However, long-term anticoagulation has been continued to prevent recurrence.

BPA under the infusion of PDE-III inhibitor and taking dual pulmonary vasodilators may be effective for such CTEPH patient with haemodynamic instability.

## Data Availability

The data underlying this article will be shared on reasonable request to the corresponding author.

## Notes

**Consent:** The patient gave consent to appear in the publication in accordance with the COPE guidelines.

**Funding:** None declared.

